# Toward Responsible and Sustainable Data Sharing in Large-scale Cohort-based Genomic Research

**DOI:** 10.1093/gpbjnl/qzaf100

**Published:** 2025-11-06

**Authors:** Jie Song, Wenwen Chen, Jin Huang, Huan Song

**Affiliations:** West China Biomedical Big Data Center, West China Hospital, Sichuan University, Chengdu 610041, China; Med-X Center for Informatics, Sichuan University, Chengdu 610041, China; West China Biomedical Big Data Center, West China Hospital, Sichuan University, Chengdu 610041, China; Med-X Center for Informatics, Sichuan University, Chengdu 610041, China; Department of Urology, Innovation Institute for Integration of Medicine and Engineering, Lab of Health Data Science, West China Hospital, Sichuan University, Chengdu 610041, China; West China Biomedical Big Data Center, West China Hospital, Sichuan University, Chengdu 610041, China; Med-X Center for Informatics, Sichuan University, Chengdu 610041, China

Since the first genome-wide association study (GWAS) was published in 2005 [1], enormous progress has been made in elucidating the genetic underpinnings of common complex diseases or traits over the past two decades. While earlier GWAS studies mainly use cross-sectional case-control designs to identify disease-associated single nucleotide variants, the emerging large cohorts with available genomic data, such as population-based biobanks (*e.g.*, UK Biobank [2], iPSYCH [3], and deCODE [4]), have opened new avenues for exploring complex quantitative traits among deeply phenotyped participants. The specific advantages of these resources are shaped by the design, depth, and diversity of the cohort. Longitudinal cohorts with repeated measures over time, in particular, provide unique opportunities to investigate phenotypes that require temporal resolution, such as disease trajectories [5], preclinical biomarkers [6], and gene–environment interactions [7]. Furthermore, those datasets enable a wide range of downstream applications, including the development of polygenic risk scores [8], causal inference using Mendelian randomization [9], the identification of early intervention windows [10], pharmacogenomic discovery [11], and the validation of potential therapeutic targets [12–14], thereby offering a powerful foundation for precision medicine and translational research.

Despite the decreasing costs of genotyping and sequencing technologies, the rapid expansion of cohort sizes and the growing demand for multi-omics data, such as transcriptomics, epigenomics, and metabolomics data, continue to make cutting-edge genomic research financially and logistically unfeasible for individual researchers or small research teams. As a result, there is a prompt increasing call for open and collaborative data sharing across the scientific community. However, in practice, only a limited number of cohort databases currently have well-established data sharing policies. While data sharing is often perceived primarily as a matter of principal investigators’ willingness, the reality is far more complex. Issues such as ethical and legal constraints, data privacy concerns, institutional policies, a lack of standardized protocols, financial sustainability, potential conflicts over authorship, and intellectual property all present significant barriers to effective data sharing.

Recent reviews have provided valuable overviews of technical, ethical, and governance challenges as well as future directions in genomic data sharing worldwide [15–18]. In this perspective paper, we draw upon our first-hand, practical experiences as cohort initiators to highlight the multifaceted challenges encountered during the actual implementation of cohort-based genomic data sharing in China. As a complement to prior discussions, we aim to shed light on the structural, operational, and financial barriers that persist in a specific regional context and to outline actionable steps toward establishing a more open, responsible, and sustainable genomic data sharing ecosystem. **Table 1** provides a summary about the main challenges and actionable proposals we have discussed.

**Table 1:** Key challenges and actionable proposals for genomic data sharing from cohort initiatives

Category	Challenges	Cross-cohort/country practices	Actionable recommendations toward sustainable ecosystem
Financial cost	Building and maintaining genomic databases require continuous investment in sequencing, storage, curation, and follow-up Limited funding often deprioritizes external data sharing	UKB (tiered access fees + cloud computing costs + subsidies for early-career researchers or those from low/middle-income countries) All of Us (no access fee but user-borne computational costs)	Introducing tiered cost-sharing or subscription-based access models Encouraging joint funding across institutions Offering subsidized or grant-based access for underfunded researchers to ensure equity
Infrastructure and technical capacity	Data integrity and sustainability requires secure biobanks, cloud servers, data management systems, and standardized analytical pipelines Lack of interoperability limits collaboration	UKB and All of Us rely on centralized cloud infrastructures Nordic cohorts use federated models to promote collaboration while reserving data sovereignty GA4GH provides models for federated access and data-use harmonization	Developing or applying federated data-sharing infrastructures Adopting interoperable data standards Investing in training and technical support for data managers
Ethical, legal, and regulatory concerns	Differences in data protection laws complicate cross-border sharing Unresolved questions on return of genomic results and dynamic consent persist	GDPR (EU and UK): strict data export controls PIPL (China): Data localization requirements and strict assessment of cross-border data transfer	Implementing dynamic consent frameworks Using privacy-preserving technologies (*e.g.*, federated analysis and on-site terminals) Aligning data-sharing protocols with national and international laws
Governance and fair access	Conflicts may arise regarding data access, authorship, and research priority Lack of transparent governance deters collaboration	Governance committees (*e.g.*, UKB Access Board) oversee data-use proposals	Establishing governance committees for data access approval Requiring project proposals to ensure ethical and scientific merit Maintaining internal visibility of approved projects to avoid duplication
Intellectual property and benefit-sharing	Disputes over ownership of derived discoveries hinder openness	H3Africa developed authorship and benefit-sharing frameworks linked to contribution levels	Defining ownership of “foundational” *vs*. “derivative” discoveries Standardizing co-authorship and benefit-sharing policies Promoting transparency in intellectual property agreements and licensing models Engage funding agencies and journals to incentivize openness and equity


*Note*: UKB, UK Biobank; GA4GH, Global Alliance for Genomics and Health; GDPR, General Data Protection Regulation; PIPL, Personal Information Protection Law; H3Africa, Human Heredity and Health in Africa.

## Challenges in genomic database construction and maintenance

The key challenges in implementing large-scale genomic data sharing from cohort studies include but not restricted to: (1) prohibitive financial costs for database construction and maintenance, (2) significant infrastructure and technical demands for data storage, standardization, and interoperability, and (3) complex ethical and legal hurdles regarding data governance. To illustrate these challenges more concretely, we outline several major obstacles we have faced below, drawing from our practical experiences.

### High financial costs for sustainability

One of the primary barriers to open data sharing is the substantial cost of genomic database construction and maintenance, which encompasses not only initial cohort recruitment and phenotypic collection but also ongoing data generation, storage, and curation.

First, high-quality genomic research often requires large sample sizes to ensure statistical power, as does deep phenotypic characterization, which often involves well-structured multidimensional measures, longitudinal follow-ups, and linkages with external databases (*e.g.*, electronic medical or health records). Therefore, balancing the breadth and depth of both genomic and phenotypic data within limited budgets remains a core challenge.

Second, unlike conventional databases, genomic datasets are massive, requiring scalable cloud storage solutions, high-performance computing, and continuous software updates. Additionally, trained personnel, including IT security experts and administrative staff, are necessary to manage data integrity, security, and accessibility. Therefore, the operational costs are substantial.

Therefore, a high-standard genomic cohort database requires sustained and considerable investment in both financial and human resources. In reality, the costs associated with follow-up visits, multi-omics profiling, and maintaining participant engagement frequently exceed initial projections. Many cohort developers struggle to secure ongoing funding to support database maintenance, which often pushes data sharing with external researchers to a lower priority amid competing demands for limited resources.

### Infrastructure and technical capacity

Following cohort recruitment, data storage and quality control are all non-negligible and important parts of cohort establishment. The infrastructure includes but is not restricted to biobanks (for sample storage), hardware/servers (for phenotype and omics data storage), and platforms/frameworks (for sample tracking, secure data sharing, multi-site collaboration networks, and ethical compliance systems). Technical capacity encompasses expertise in multi-omics assay analyses, robust standardized protocols, computational skills for software maintenance, and analytical skills for data processing and quality control. All of the above entails both human and material resources to ensure data integrity and sustainability and must be borne in mind for cohort development and maintenance. The cohort initiators need to make great efforts toward such training and management.

In addition to hardware and computational resources, standardization and interoperability are equally crucial for effective data sharing. Commonly, centralized data pooling is neither feasible nor desirable due to technical inconsistencies, privacy concerns, and data sovereignty barriers. It remains a major challenge to establish and coordinate common data and technical standards across diverse data sources.

### Ethical, legal, and regulatory concerns

The collection and sharing of genomic data raise complex ethical issues. As sequencing technologies improve and more genetic variants become detectable, the question of which results and to what extent the interpretations should be returned to participants remains an unresolved issue [19]. Ethical principles such as non-discrimination, participant autonomy, transparency, and personalized benefits should always be borne in mind [20], alongside emerging needs for dynamic consent, a flexible, ongoing process that allows participants to modify their consent preferences as research goals evolve.

Moreover, sharing data across different regions, especially between countries, remains a significant challenge due to varying regulations on individual privacy and data protection. Data-sharing frameworks differ substantially across countries. For example, the UK Biobank [2] operates under the General Data Protection Regulation (GDPR) and a framework of central governance; China’s Personal Information Protection Law (PIPL) imposes additional layers of data localization and export approval; Nordic biobanks adopt federated data systems; and the US initiatives such as TOPMed [21] promote cloud-based access. Consequently, project collaboration brings extra work for paperwork (*e.g.*, ethical protocols), data management, and ethical considerations. For example, data harmonization requires the sharing of analysis pipelines, including standardized quality control procedures and the imputation of data generated from different genotyping arrays. However, owing to the European Union’s GDPR, genetic data from European countries cannot be uploaded to external platforms such as the US-based TOPMed imputation server [21] without specific ethical approval. Understanding these contrasts highlights the need for adaptable governance frameworks tailored to national legal environments.

## Steps on the way to sharing genomic data from a cohort database

Despite these many challenges, we acknowledge that sharing data with external users, both domestically and internationally, is an essential step for maximizing the scientific and societal value of genomic cohort studies.

However, we argue that effective data sharing cannot be achieved solely by encouraging principal investigators to “release” their data. Rather, a more sustainable and collaborative model must be established: one in which prospective data users are invited to participate meaningfully in the cohort ecosystem. This could include contributing funding or technical expertise, complying with data governance frameworks, and sharing resulting data or analytic tools back with the community.

In addition to calling for increased attention and support from governmental bodies, we propose several actionable strategies to ensure the safe, sustainable, and equitable management of genomic data in a multi-user environment, drawing from our perspective as cohort initiators. This includes the establishment of clear policies and regulatory frameworks, along with a well-structured management system, which we view as a critical foundation for enabling meaningful, secure, and responsible data sharing.

### Achieving financial sustainability for database maintenance

A viable solution to address the financial challenge is to implement a structured cost-sharing model among users, where access fees are tiered based on data complexity and resource use. Alternatively, institutional collaborations could distribute financial burdens by pooling resources among multiple research entities.

For instance, the UK Biobank Research Analysis Platform (UKB-RAP) [22] has pioneered secure access to large-scale individual-level data without the need for data movement. Researchers using UKB-RAP are responsible for computational costs in addition to the data access fee. Similarly, the All of Us Research Program in the United States employs a cloud-based Researcher Workbench with a Controlled Tier for data access [23]. Unlike the UK Biobank, only registered researchers from institutions that have data use and registration agreements with All of Us covering the Controlled Tier are eligible to access genomic data. Consequently, there is no cost for data access, but researchers need to incur costs for computation and data storage.

However, a balance between financial sustainability and equitable access is critical, as high costs could limit participation, particularly for early-career researchers, or those from underfunded institutions. Transparent financial policies and long-term funding strategies, ideally together with efforts from data users, must be established to ensure that the database remains operational and accessible. The UK Biobank provides financial support packages for students and researchers at early stages or from low- and middle-income countries, including subsidized access tiers and dedicated grants from industry. Including such models demonstrates the scalability of cost-sharing mechanisms and their real-world impact on research efficiency, while balancing sustainability with inclusivity.

### Ensuring data security and interoperability within legal frameworks

Genomic data are both highly sensitive and scientifically valuable, making them a prime target for misuse or cyberattacks. Even de-identified datasets can potentially be re-identified when cross-referenced with other sources. Robust security protocols — including encryption, multi-tiered access control, regular audits, and cybersecurity infrastructure — are essential.

Compared with centralized cloud-based models for aggregated databases, the federated data sharing framework might be more applicable for cohort initiators in China. It enables individual data custodians to maintain their own databases while facilitating cross-institutional access through interoperable standards. Analytical workflows in this setting can be implemented in two ways: independent analyses of individual datasets with subsequent result aggregation, or temporary integration of relevant data subsets within a shared computational environment for joint analysis, where permitted. Such infrastructure supports remote querying capabilities to address specific research questions. Internationally, emerging frameworks such as Data Use Ontology [24,25] and Beacon protocols [26] developed by members of Global Alliance for Genomics and Health (GA4GH) [27] provide practical pathways toward harmonized data access across institutions, which enhances cross-cohort comparability and facilitates federated analysis.

For cross-border collaboration between China and other countries, federated systems or data visiting models compliant with international regulations such as GDPR and PIPL are essential. The Nordic Tryggve infrastructure illustrates how container-based platforms can support secure, compliant cross-border data sharing for multi-institutional research [28]. Similarly, for Chinese cohorts, on-site secure analysis terminals or remote code submissions with local execution could be adopted to balance data accessibility and regulatory compliance with PIPL. In practice, GWAS summary statistics could be uploaded to Genome Sequence Archive for Human (https://ngdc.cncb.ac.cn/gsa-human) [29] after conforming to the regulations of the Human Genetic Resources Administration of China. Moreover, analyses could be conducted under the jurisdiction with stricter regulatory requirements to ensure compliance on both sides.

### Managing fair access and preventing research conflicts

As a genomic database expands its user base, conflicts may arise among different research groups in terms of data access, priority research areas, and publication rights. Internal teams may feel that they should have privileged access to the database, while external collaborators may demand equal opportunities to utilize the resource. If not properly managed, these tensions can hinder collaboration, slow research progress, and even lead to disputes over scientific contributions.

One solution is to establish a governance committee, including cohort initiators and domain experts from different disciplines, responsible for overseeing data access and resolving conflicts. The committee can implement a standardized application process. Researchers are required to submit a project proposal as part of their data access application to ensure that the use of the database is aligned with its scientific objectives and ethical standards. Each proposal should include clear research aims, analytical plans, anticipated benefits, and data management strategies. The committee review board will evaluate the proposals and ensure that data access is granted based on scientific merit, feasibility, and relevance rather than institutional affiliation or seniority. Approved projects could be made internally visible to data users to prevent duplication and encourage collaboration among teams with overlapping interests. Furthermore, rules regarding data embargo periods, co-authorship expectations, and dispute resolution mechanisms should be clearly outlined in data use agreements. A well-defined governance framework helps maintain transparency, promotes collaboration, and minimizes conflicts that could otherwise hinder the potential impact of the database.

### Establishing fair intellectual property and benefit-sharing policies

A major concern in open-access genomic research is how to fairly allocate intellectual property rights and distribute benefits arising from shared data. While users contribute to the research process by analyzing data and generating findings, the database itself represents years of investment in participant recruitment, data collection, storage, and management. Without clear policies, tensions may arise regarding who owns the rights to new discoveries, such as biomarkers, disease risk prediction models, or drug targets derived from the database. To prevent such disputes, a well-defined intellectual property and benefit-sharing framework should be established before granting access to external users.

One approach is to classify different types of outputs — such as publications, patents, and commercial applications — and specify the rights and obligations of both parties. This includes defining key terms, where foundational discoveries refer to core scientific findings that directly result from the initial establishment of the database, including fundamental datasets, baseline analyses, and generalizable insights that underpin subsequent research. Agreements can stipulate that the database initiators/organizers retain primary rights to these foundational discoveries, while external users may be granted rights to specific research outputs developed through their analyses, depending on the extent of their contribution.

Additionally, benefit-sharing mechanisms, such as co-authorship requirements, licensing agreements, or financial returns from commercialized findings, can be implemented to ensure fair distribution of research benefits. Existing initiatives, such as Human Heredity and Health in Africa (H3Africa) [30], have developed benefit-sharing frameworks that link authorship and data ownership to measurable contributions. Adapting similar guidelines, *e.g.*, prioritizing data availability for data-generating investigators, requiring co-authorship for substantial data provision, or standardizing benefit-sharing templates, can prevent disputes and clarify expectations. Establishing these policies in advance will not only protect the database organizers’ long-term interests but also foster a transparent and collaborative research environment.

## Conclusion

This perspective paper highlights the transformative potential of genomic data from large cohort studies in advancing biomedical research and public health, while recognizing the significant challenges involved in constructing, maintaining, and sharing such datasets. Drawing from our own experiences, we outlined key concerns for sharing genomic data from cohort databases, such as data security, financial sustainability, governance, and intellectual property rights. While our recommendations represent one approach, we acknowledge that alternative strategies may be equally valid and should be explored in future work.

Above all, we argue that clear policies and structured management frameworks are vital for effective and responsible data sharing. Equally important is fostering awareness among data users of their role in supporting and sustaining the growing database — whether through funding, technical contributions, or reciprocal data sharing. This shared responsibility is crucial for building a more sustainable, collaborative model and strengthening the overall cohort ecosystem.

Looking forward, future efforts should focus on the development of universal data-sharing standards, interoperable federated access systems, and policy advocacy among funding agencies and journals to incentivize openness and equity. In parallel, documenting and sharing best practices, as well as integrating centralized efficiency with federated compliance, may help establish a sustainable hybrid model for emerging cohorts.

In summary, the construction and sharing of genomic databases built upon large cohorts hold immense promise. However, realizing this potential requires collective effort and shared responsibility, to overcome the practical, ethical, and financial challenges involved, and to fully unlock the value of these resources for scientific discovery and societal benefit.

## CRediT author statement

**Jie Song:** Conceptualization, Writing – original draft, Writing – review & editing. **Wenwen Chen:** Writing – original draft. **Jin Huang:** Supervision, Writing – review & editing. **Huan Song:** Conceptualization, Supervision, Writing – original draft, Writing – review & editing. All authors have read and approved the final manuscript.

## Competing interests

All the authors have declared no competing interests.
